# Temperature Scanning Stress Relaxation of an Autonomous Self-Healing Elastomer Containing Non-Covalent Reversible Network Junctions

**DOI:** 10.3390/polym10010094

**Published:** 2018-01-19

**Authors:** Amit Das, Aladdin Sallat, Frank Böhme, Essi Sarlin, Jyrki Vuorinen, Norbert Vennemann, Gert Heinrich, Klaus Werner Stöckelhuber

**Affiliations:** 1 Leibniz-Institut für Polymerforschung Dresden e.V., Hohe Straße 6, 01069 Dresden, Germany; das@ipfdd.de (A.D.); sallat@ipfdd.de (A.S); boehme@ipfdd.de (F.B.); gheinrich@ipfdd.de (G.H.); 2 Laboratory of Materials Science, Tampere University of Technology, P.O. Box 589, 33101 Tampere, Finland; essi.sarlin@tut.fi (E.S.); jyrki.vuorinen@tut.fi (J.V.); 3 Faculty of Chemistry and Food Chemistry, Department of macromolecular Chemistry, Technische Universität Dresden, D-01062 Dresden, Germany; 4 Faculty of Engineering and Computer Science, University of Applied Sciences Osnabrück, 49076 Osnabrück, Germany; n.vennemann@hs-osnabrueck.de; 5 Institut für Textilmaschinen und Textile Hochleistungswerkstofftechnik, Technische Universität Dresden, D-01062 Dresden, Germany

**Keywords:** self-healing, bromo-butyl rubber, ionic modification, reversible polymer network, stress-relaxation

## Abstract

In this work, we report about the mechanical relaxation characteristics of an intrinsically self-healable imidazole modified commercial rubber. This kind of self-healing rubber was prepared by melt mixing of 1-butyl imidazole with bromo-butyl rubber (bromine modified isoprene-isobutylene copolymer, BIIR). By this melt mixing process, the reactive allylic bromine of bromo-butyl rubber was converted into imidazole bromide salt. The resulting development of an ionic character to the polymer backbone leads to an ionic association of the groups which ultimately results to the formation of a network structure of the rubber chains. The modified BIIR thus behaves like a robust crosslinked rubber and shows unusual self-healing properties. The non-covalent reversible network has been studied in detail with respect to stress relaxation experiments, scanning electron microscopic and X-ray scattering.

## 1. Introduction

Nature has always been an ultimate inspiration for the development of new technologies. Over a span of millions of years, biological evolution has perfected the art of self-healing, to ensure that a species was fit enough to survive and life continued. The way a lizard can regrow its limb, or animals and humans can repair wounds, have always been a matter of great interest. What if such capabilities could be incorporated into technology? What if materials could heal themselves? Inspired from this, development of self-healing materials has emerged to be a challenging area to control damage to a lifeless object (see i.e., [1]).

Due to enormous advantages like light weight, durability, resistance against natural weathering and easy processability, synthetic polymer composites are gradually replacing conventional materials. High performance polymer composites equipped with self-healing properties would be the ultimate materials of choice for different high-tech applications [2].

The propagation of micro-cracks may affect the structural integrity of polymeric components, for example tires, leading to catastrophic failure. Therefore, a concept of self-healing polymeric materials was proposed in the 1980s [3] as a means of healing invisible micro-cracks for extending the lifetime and the safety of these materials. Most of the self-healing work has been done on brittle, thermosetting polymers [4,5] and high performance fibre-reinforced epoxy composites [6]. In these cases, the basic principle of self-healing mostly follows two principles. Majority of the studies has been done on supplying the curatives to the damaged zone by capsules or by micro-channel feeding. In these methods, some liquids are allowed to solidify in the damaged area and, consequently, the healing takes place. The kinetics of the curing reaction is very important which can be influenced by the healing conditions. Much literature can be found where different types of cross-linkable monomer and pre-polymers are used to heal the defects [7,8,9,10,11,12]. For examples, epoxies, cyanoacrylates/cyanoacrylates, polyols along with isocyanates, some monomers with ring opening polymerization tendency and organic molecules which are susceptible to click reaction are the most studied self-healing components applied in polymeric composites. Usually, external or internal stimuli are necessary to fill the crack with the healing agent. The fabrication or the design of a self-healing material will be only successful if the healing process starts immediately after the material experiences damage. Otherwise, there would be a premature reaction which is not desirable. A couple of favourable principles could be realized to overcome such undesirable reactions. Firstly, the reactive components could be kept inside a capsule and when necessary it could be exposed to the damaged area. Secondly, the reactive curatives could be allowed to pass through the micro channels to the damaged area immediately after the incident. For systems containing self-healing capsules the repeatability of the process is limited as the capsules, once supplied the reactive curatives, will be damaged and there will be no possibility to refill the capsule. In this respect, the micro-channel feeding methods are more applicable in multiple healing.

Another principle of self-healing is based on the mechanism involved with self-association or self-aggregation of the polymeric materials [8,13]. The ideal choice of self-healing materials should mimic the self-healing principle of biological systems. Therefore, bonds between the molecules which undergo dissociation/breakage during crack and damage should be reformed again after having a signal or stimuli. For this purpose, association of the molecules or formation of new bonds could be realized by hydrogen bonds, ionomers, π–π interactions and metal coordination, or by weak covalent bonds. So, reversibility of the network structure is the main criteria for intrinsically self-healing materials. Here, it could be mentioned, though sulphur-sulphur bonds are reversible and most of this reversible character has been found in biological systems, for solid-state sulphur crosslinked rubbers the reversibility of sulphur-sulphur linkages has not yet been conclusively proven [14].

A raw rubber polymer without any crosslinking behaves like a viscous material and the direct application of this material to engineering fields is rather limited. Owing to the viscous character this rubber behaves like an intrinsic self-healing material following the classical polymer reptation phenomenon [15]. The self-healing tendency will completely disappear after the raw rubbers are treated with crosslinking agents. The crosslinking step transforms the rubber into a semi-solid elastic material with higher strength, better toughness, enhanced hardness and modulus. Crosslinking transform the annealed polymer system into a quenched system (polymer network), if these processes are discussed within a more fundamental viewpoint of statistical thermodynamics [16,17]. The established polymer network structures lead to restricted mobility of the macromolecular chains. Usually, permanent network structures of the raw rubbers are established by covalent crosslinking using sulphur or peroxide [18]. However, supramolecular assembly of relatively small molecules could also lead to the formation of chains and crosslinks and thus highly elastic materials can be designed. Furthermore, polymeric compounds with ionic clusters as crosslinking sites can even be physically mixed with commercial rubber to develop rubber compounds with self-healing properties [19]. Similarly, a network node could be established by introducing ionic functionalities in the macromolecular chemical structure which eventually results into a three-dimensional network structure by the virtue of ionic association. In our previous work a self-healing character of ionically modified bromo-butyl rubber was reported [20]. It was found that the ionic cluster formation was facilitated at 100 °C and, consequently, the self-healing process can be accelerated at this temperature. Therefore, ionically modified BIIR was filled with conducting carbon nanotubes leading to a sufficient conductivity of the rubber matrix which can be then heated by implying electrical voltage [21]. This modified BIIR was also used as a blend component, together with Natural Rubber (NR), where the carbon nanotubes act additionally as reinforcing filler within the blend matrix [22].

In this study, we investigated the stress relaxation characteristics of modified self-healing bromo-butyl rubber which was finally compared with the corresponding standard sulphur cured sample. Stress relaxation properties of rubbers are of extraordinary importance concerning the performance of these kinds of soft materials under several service conditions (see, e.g., [23,24]). This self-healing nature of the material is further elucidated by scanning electron microscopic studies, creeping experiments and X-ray scattering.

## 2. Materials and Methods

In this study, the bromo-butyl rubber was kindly provided by Lanxess (LANXESS International SA, Granges-Paccot, Switzerland; Bromo-butyl X2, Bromine Content 1.8%, Mooney Viscosity). Sulphur (99.50%, sublimed), magnesium oxide (98%), zinc oxide from Acros Organics (Acros Organics, Geel, Belgium) and stearic acid (general purpose grade) from Fisher Scientific (Fisher Scientific UK, Loughborough, UK) were used in the study. 2-Mercapto-Benzothiazol used as organic accelerator was of industrial grade.

### 2.1. Preparation of an Imidazolium-Based Ionomer

The preparation of the imidazolium-based ionomers was done by N-alkylation of 1-butyl imidazole with commercial bromo-butyl rubber. The reaction between BIIR with butyl imidazole was carried out using internal mixer Thermo Haake Rheocord PolyLab 300p (Thermo Electron, Karlsruhe, Germany) with 10 min mixing time. This reactive mixing was done at 85 °C with a rotor speed of 60 rpm. This compound was then compression moulded at 100 °C for 10 min. For comparison, a sulphur cured vulcanizate was prepared by compounding MgO (0.5 phr), stearic acid (1 phr), sulphur (0.5 phr), zinc oxide (3 phr) and 2-mercaptobenzothiazole (1.5 phr) with the rubber. The mixing was carried out using two-roll mixing mil (Polymix-110L, Servitec GmbH, Wustermark, Germany) at 30 °C with a constant friction ratio of 1:1.2.

### 2.2. Tensile and Creeping Experiments

The tensile test was carried out by a material testing machine (Zwick 1456, Z010, Ulm, Germany) at dumbbell shaped specimens (S2 size) with a with a crosshead speed of 200 mm·min^−1^, using optical strain control (according to DIN EN ISO 527-2/S2/200). The creep recovery test was performed using the same instrument.

### 2.3. X-ray Scattering 

The Small-Angle X-ray Scattering (SAXS) measurements were carried out with a Panalytical Empyrean diffractometer (Malvern Panalytical B.V., Almelo, The Netherlands) using Cu Kα radiation. The diffraction profiles were acquired in the angular range of 0°–5° (angle 2θ) with step size of 0.01° and step time of 29 s per step.

### 2.4. Temperature Scanning Stress Relaxation (TSSR)

A constant tensile strain—i.e., 50%—was applied to the sample for two hours to allow the preconditioning of the samples before applying further stress. For this measurement standard (S2) dumbbell shaped specimen with ~2 mm thickness was used. After two hours pre-strain the sample was heated linearly with a rate of 2 K/min till the sample failed. The test was done with a commercially available TSSR instrument (Brabender GmbH, Duisburg, Germany) [25,26,27].

A more detailed characterization of the imidazole modified bromo-butyl rubber system, including DSC measurements and in-depth DMA-studies can be found in our previous paper [20].

## 3. Results and Discussion

**Figure 1:**
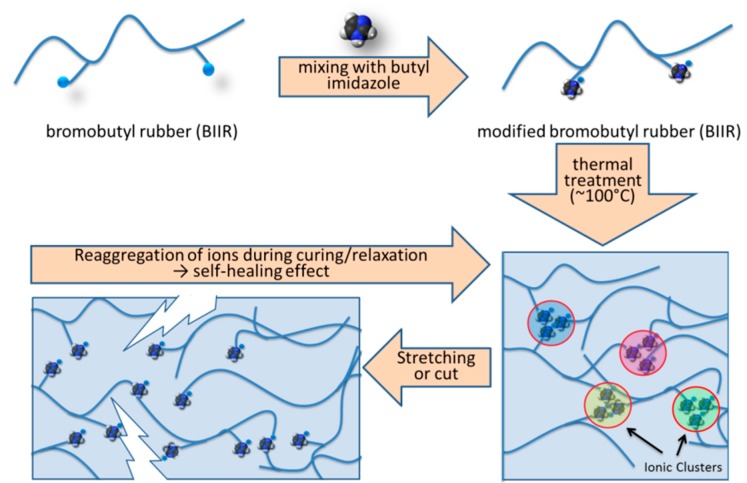
Schematic presentation of modification of bromo-butyl rubber (BIIR) by butyl imidazole. The ionic imidazolium bromide group can be associated together to form a crosslinked-type network structure. This network is broken during damage and rebuild during healing.

**Figure 2:**
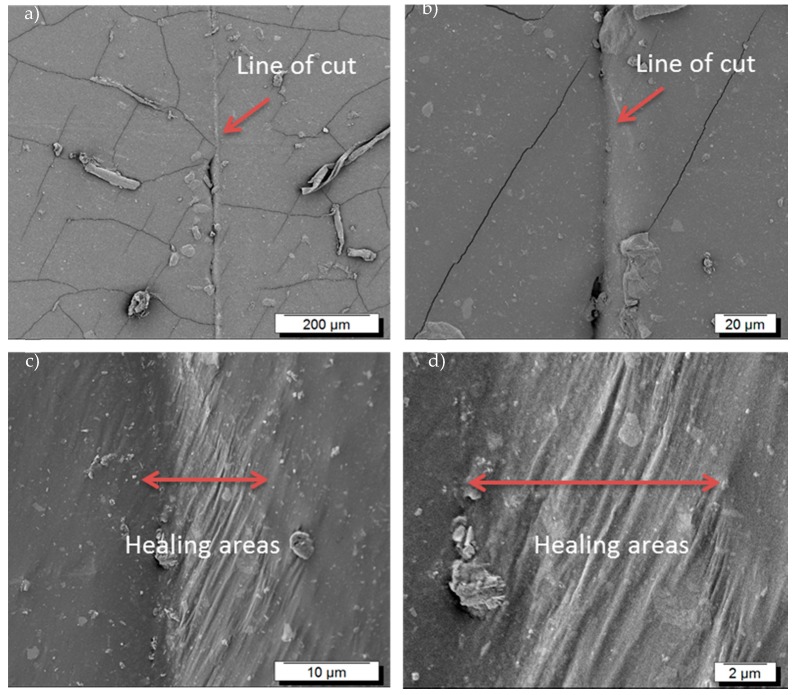
Scanning electron microscopic pictures of imidazole modified BIIR rubber. The samples were cut into two pieces (**a**) and allowed to heal for 24 h (**b**–**d**).

Figure 1 describes the self-healing mechanism of the modified BIIR and the details can be found in our previous publication [20]. Here, the self-healing behaviour was verified by direct microscopic investigation. In this experiment, a modified rubber sample was cut into two pieces and subsequently joined together and kept for 24 h for healing. Figure 2 shows the SEM images of the surface of a sample healed after a healing period of 24 h at room temperature. It is clear from these images that the cut is almost repaired. After healing, a clear mark is still prominently visible along the line of damage. Mechanical cut can make an incision to both clusters as well as the polymer chains and due to this fact, a complete healing of the cut is not possible. However, a closer look at this cut shows that the sharp line of cut is almost filled by the polymers.

At the same time, some permanent cracks and holes could be also found along the cut line. Beside the damaged areas (line of cut) some other fine cracks were also observed throughout the surface. Obviously, these fine cracks arise due to the sensitive nature of the butyl rubber chains towards high energy electron irradiations exposed during scanning electron microscopy imaging and preparation of the sample by gold sputtering technique. This is already known and typical in butyl rubber [28]. Nevertheless, the matrix has significant ability to heal the main cut.

**Figure 3:**
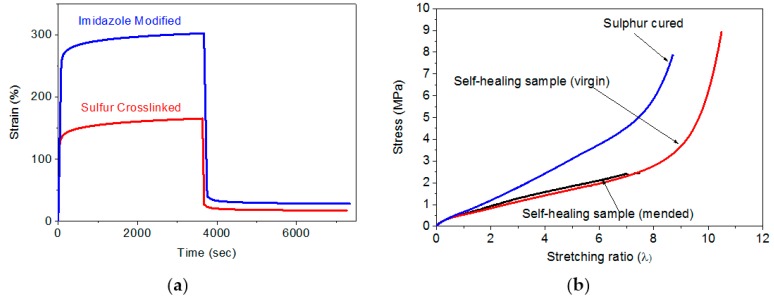
(**a**) Stretching experiment of the modified BIIR and the mended sample. The mended sample was previously cut half of the width and allowed to heal for 1 h; (**b**) Creeping experiment of the sulphur vulcanized and modified BIIR rubbers.

This intrinsic healing character appeared due to the ability of re-association of the ionic groups leading to re-clustering of the ions (Figure 1). Figure 3a shows the stress-strain plots of the modified and the sulphur cured rubber. The nature of the ionically modified BIIR curve resembles that of a crosslinked elastomer though the modified rubber was not permanently crosslinked by peroxide or sulphur vulcanization ingredients. However, the ultimate tensile strength and elongation at break of the ionically modified rubber is superior compared to the traditional sulphur cured sample. The mended sample did not perform like an undamaged sample as the tensile strength and elongation at break values were lower. This can be associated with the permanent cleavage of rubber chains by mechanical cut. The modified BIIR and sulphur cured BIIR were further investigated with creeping experiments to understand the elastic nature of the matrix. Figure 3b displays the results of the creep test performed at room temperature. A constant stress of 2 MPa was applied to the dumbbell shaped specimens and after 1 h the strain obtained for sulphur crosslinked and imidazole modified samples was ~165% and ~302%, respectively. The higher strain of the modified rubber is ascribed to the lower mechanical stiffness compared to the sulphur cured rubber. After releasing the applied stress the permanent set was found to be 18% and 28%, respectively, at the end of 1 h relaxation time. The larger value of residual strain for modified sample could be attributed to the ion hopping mechanism during stretching. One ion pair can be slipped from one cluster to another cluster due to the less strained configuration.

**Figure 4:**
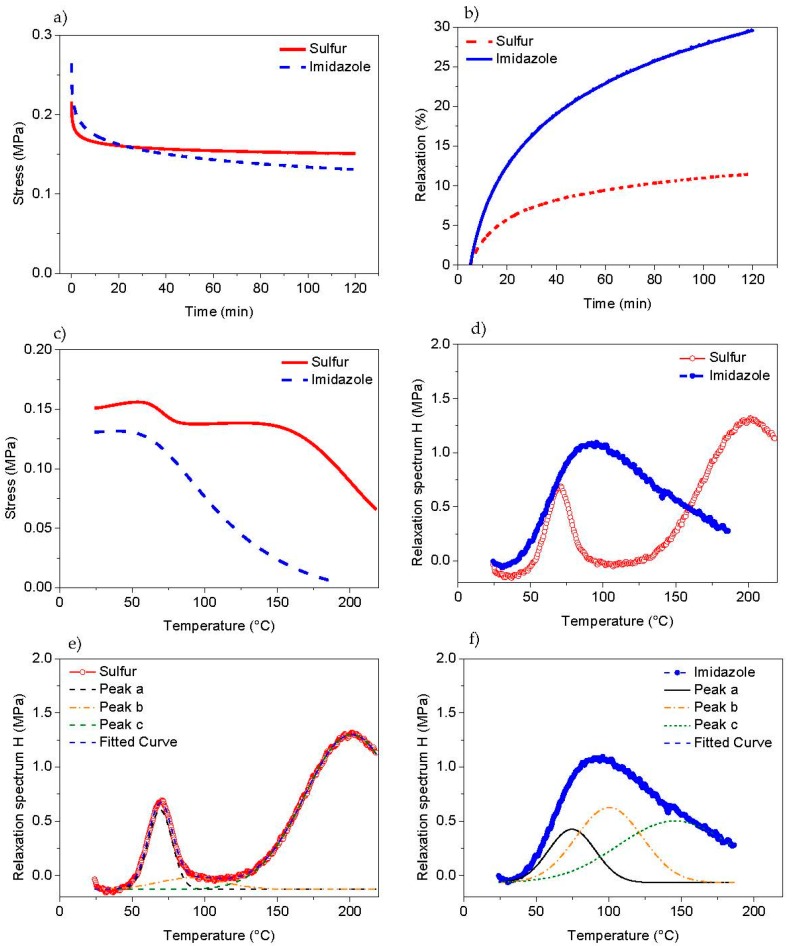
Isothermal relaxation test at 23 °C; (**a**) stress at constant strain (stretching ratio λ = 1.5); (**b**) stress relaxation as a function of time. Non-isothermal relaxation test; (**c**) stress vs. temperature curves and (**d**) relaxation spectra. De-convoluted relaxation spectra of (**e**) sulphur cured BIIR and (**f**) imidazole modified BIIR.

To better understand the crosslinking process associated with the ionic groups, temperature scanning stress relaxation (TSSR) [25,26,27] experiments were done (Figure 4). According to the simplest (neo-Hookean) theory of rubber elasticity the mechanical (nominal) stress can be expressed by σ = ρ*RT*⁄*M*_C_ (λ − λ^−2^) where ρ is the density, λ = *L*/*L*_0_ is the stretching ratio, *R* is universal gas constant and *M*_C_ is the average molar mass of the network chains. According to the above relation the stress is proportional to temperature at a certain constant stretching ratio. Thus, the temperature coefficient of the stress can be written as *k* = (*d*σ⁄*dT*)_λ_ = *νR*(λ − λ^−2^). At lower stretching ratio (λ < 1.07) the value of *k* is negative but at higher stretching ratio the value is positive due to the entropic elastic nature of the rubber. The change from a negative to a positive temperature coefficient is often referred to as thermoelastic inversion. The reason for the negative coefficient at small strains is the positive thermal expansion and that the force-temperature curves are obtained at constant length. An increase in temperature causes thermal expansion (increase of the initial length of the specimen *L*_0_) and consequently a decrease in the strain ratio λ = *L*/*L*_0_ at constant *L* [29]. In the TSSR test, the sample is kept at constant strain, i.e., λ = 1.5 and the temperature is raised at constant rate. Therefore, the stress relaxation modulus can be studied with respect to temperature and the corresponding relaxation spectrum will be given as *H*’ = −*T*(*dE*_non-iso_(*T*)⁄*dT*)_at const.heating rate_, where *E*_non-iso_ is the non-isothermal relaxation modulus as a function of temperature.

The results of the isothermal relaxation measurements (at 23 °C) are presented in Figure 4a,b. It becomes obvious, that the imidazole modified BIIR exhibits much stronger stress relaxation than the sulphur cured sample. The reason can be a lower crosslink density of imidazole modified BIIR. It should be mentioned here that in the present case BIIR is not conventionally crosslinked by externally added curatives but the crosslinking character originates solely from the ionic association of imidazole groups. In the case of imidaziole modified BIIR, the network is strong enough to give the material a certain mechanical strength comparable with covalent crosslinked networks. At the same time, a sufficient dynamic of the network facilitates healing effects. This specific kind of network dynamics can be attributed to the diffusion of a primitive chain through slip-links which represent the presence of entanglements and considers additionally the presence of weakly associating reversible cross-linkers on this kind of diffusion. A theoretical in-depth discussion of the physics of such a network with of weak reversible cross-linkers can be found in [30]. Hereby, the situation in imidazole modifies BIIR is somewhat different from transient polymer network undergoing small deformations, based on the rate of breaking and reforming of network cross-links and the evolving elastic reference state (as i.e., described in [31]) on the whole, the dynamics reflected in these systems is certainly more complex, also involving distributions of ionic cluster sizes and bond lifetimes. This topic has been recently studied and discussed in some more details in [32].

Due to lack of sufficient imidazole bromide groups at the backbone of the rubber chains the crosslinking efficiency is rather weak as compared with standard sulphur cured compound. A recent publication also reported that poly-isobutylene based ionic liquid exhibited a defined nano- and mesoscale ordering behaviour resulting in different viscoelastic behaviour of the rubber [33].

Figure 4c,d show the stress-temperature curves and the corresponding relaxation spectra of both samples derived from non-isothermal relaxation state, respectively. It is seen that the sulphur cured sample exhibits a stronger entropy effect in the initial part of the curve, when compared to the imidazole modified sample. This indicates a higher crosslink density of the sulphur cured sample, however, the imidazole modified sample seems to be also crosslinked. Furthermore, as far as this non-isothermal relaxation spectrum of sulphur cured sample is concerned, the network structure contains at least two different types of crosslinks, represented by two distinguished peaks at about 70 and 200 °C. The peak at 200 °C can be attributed to cleavage of sulphur bridges and/or scission of the polymer main chains.

**Figure 5:**
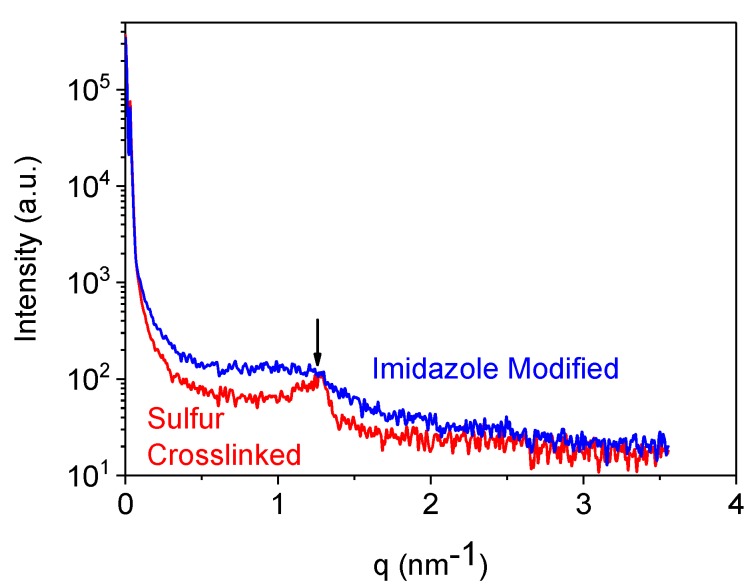
SAXS profiles of sulphur cured BIIR and imidazole modified BIIR.

The relaxation process at 70 °C is reflecting a transition of lower activation energy, possibly a weak physical interaction between two rubber chains could be behind such effect. Moreover, due to the alternative –CH_2_– and –C(Me)_2_– chemical structure of poly isobutylene chains a crystalline character of this rubber is well-known even in the un-crosslinked and strain-free state [33,34,35]. Small angle X-ray scattering experiments also show a sort of crystalline character of sulphur cured BIIR (Figure 5). In this figure, a comparatively sharp peak appeared with a corresponding length scale of ~5 nm for sulphur cured BIIR but the peak is very much broadened/disappeared for imidazole modified compound. Reasonably, the low temperature relaxation behaviour in TSSR experiment can be correlated with the melting behaviour of such crystalline structures persisted in sulphur cured BIIR.

The crystallites are acting as junctions between different polymer chains resulting in a (physical) network formation. During heating these crystallites melt and the corresponding network collapses which is reflected in the stress relaxation effect. This effect is probably dominating for the sulphur cured sample (in the range 60–90 °C) but, surprisingly, it disappears for imidazole modified samples. Obviously, ionic clustering of the imidazole groups may lead to a more heterogeneous character of the rubber chains inhibiting the crystal formation by the chains. A more detailed investigation is required to understand this stress-relaxation behaviour, particularly at lower temperatures.

In case of the imidazole modified BIIR the decay of stress starts at about 70 °C but in contrast to the sulphur cured sample no plateau region is observable. The stress slightly decreases over a broad range of temperature and approaches zero at about 180 °C. This relaxation behaviour indicates the lack of covalent crosslinks, which leads to less thermal stability of the network. To get further insight into these asymmetric relaxation peaks a de-convolution of the relaxation spectrum (Figure 4e,f) was performed by fitting the experimental data with 3 Gaussian peaks. The results show that, apart from the peak at about 70 °C, two additional peaks exist at about 100 and 150 °C. The peak at 100 °C may be attributed to the dissociation of ionic clusters and the peak at 150 °C may be associated with the degradation of the polymer main chains.

## 4. Conclusions

The presented concept of self-healing followed by ionic association of the polar groups could open a new area for further research and, furthermore, support the development of new principles for rubber design for several technical applications. Studies of mechanical relaxation spectra supported the understanding of unusual network formation of ionically modified BIIR. Besides easy processing, these materials have unique advantages over the conventional rubbers as this material does not need any curatives or crosslinking agents. Furthermore, this material could be compounded with conventional fillers like carbon black and silica for strength enhancement of the materials, if necessary. We even have shown how the described effect of autonomic self-healing without external intervention leads to significant slowing down of crack propagation in the rubber material [20], i.e., to an increase of tearing resistance. This leads to a substantially positive impact on the annual volume of dust resulting from tire wear. We are convinced that this novel approach, both from the materials and the processing point of view, may raise a significant interest in both commercial application as well as academic research.
